# Predictive Factors of Amputation in Diabetic Foot

**DOI:** 10.3390/biomedicines12122775

**Published:** 2024-12-06

**Authors:** Francesco Farine, Antonio Maria Rapisarda, Carolina Roani, Cristina Giuli, Chiara Comisi, Antonio Mascio, Tommaso Greco, Giulio Maccauro, Carlo Perisano

**Affiliations:** 1 Department of Orthopaedics, Fondazione Policlinico Universitario A. Gemelli IRCCS, 00168 Rome, Italy; francesco.farine01@icatt.it (F.F.); antoniomaria.rapisarda01@icatt.it (A.M.R.); carolina.roani01@icatt.it (C.R.); cristina.giuli01@icatt.it (C.G.); chiara.comisi22@gmail.com (C.C.); antonio.mascio87@gmail.com (A.M.); tommaso.greco2@guest.policlinicogemelli.it (T.G.); giulio.maccauro@policlinicogemelli.it (G.M.); 2 Scuola di Specializzazione in Ortopedia e Traumatologia, Università Cattolica del Sacro Cuore, 00168 Rome, Italy; 3 Dipartimento di Scienze della Vita, della Salute e delle Professioni Sanitarie, Link Campus University, 00165 Rome, Italy

**Keywords:** diabetic foot, amputation, diabetes mellitus, prevention, risk factor, prognostic factor, Hb, WBC, NLR, PLR, CRP, mortality

## Abstract

**Background**: Diabetic foot ulcers are a common complication of diabetes mellitus, often leading to progressive sensory deficits, impaired vascularization, and a predisposition to ulceration and gangrene. Untreated ulcers may cause recurrent infections that endanger a patient’s life. Amputation of part of the limb up to a well-vascularized level is one of the treatments employed for untreated ulcers. **Objectives**: Laboratory data were analyzed with the aim of identifying risk factors related to surgical amputation treatment of diabetic foot. We believe it is useful to identify risk factors that can be altered from a reversible condition in the clinical decision-making of treatment, thus manifesting themselves in a timely manner to provide the patient with an alternative to surgical amputation of the lower limb. **Methods**: Our retrospective study was conducted by the Department of Orthopedics and Traumatology at the Fondazione Policlinico Universitario Agostino Gemelli IRCCS in Rome. We recruited 200 patients who underwent lower limb amputation for diabetic foot between 2017 and 2021 and had blood tests both 30 days and within 5 days before the first surgery. **Results**: This case–control study revealed a significant negative correlation between hemoglobin (Hb) levels and the occurrence of leg amputations. In contrast, C-reactive protein (CRP) levels, the neutrophil-to-lymphocyte ratio (NLR), and the platelet-to-lymphocyte ratio (PLR) showed significant positive correlations with leg amputations. A 3-year follow-up of the cases also revealed a significative positive correlation between age, white blood cells (WBC) count, NLR, PLR, and death at 3 years. **Conclusions**: Our findings suggested that lower Hb levels and elevated CRP, NLR, and PLR levels in patients with diabetic foot disease may be associated with a higher risk of lower-limb amputation. Unexpectedly, this study also demonstrated that elderly age, WBC, CRP, NLR and PLR levels may be associated with a negative prognosis for these patients. These findings highlighted the importance of monitoring these laboratory values in diabetic patients to identify individuals at higher risk of leg amputations and implement appropriate interventions to prevent or minimize the occurrence of this severe complication.

## 1. Introduction

Diabetic foot syndrome is a serious complication of chronic diabetes, accounting for about half of non-traumatic lower-limb amputations and nearly 75% of foot amputations [1,2,3,4].

Diabetic foot ulcers (DFUs) arise from multiple factors, with peripheral neuropathy being the primary cause. Other contributing factors include peripheral vascular disease (PVD), repeated trauma, and secondary infections [5,6,7]. These factors lead to gradual loss of sensory nerve function in the feet, diminished blood supply, and an increased risk of ulcers and gangrene in untreated diabetic patients.

A diabetic foot can present itself as an ulcer, an infection, or a Charcot neuro-osteoarthropathy deformity. Diabetes mellitus is a leading cause of Charcot arthropathy, with prevalence rates ranging from 0.8% to 7.5% [8,9,10].

The impact of diabetes and DFUs on society is significant, as longer life expectancies have contributed to their increasing prevalence. Diabetes now accounts for 9% of global deaths, resulting in 4 million fatalities annually [11,12].

Infected DFUs are a primary reason for extended hospital stays. Such stays result in high morbidity among diabetic patients and impose significant financial burdens on healthcare systems, particularly when additional surgeries or amputations are necessary [13,14,15]. The level of amputation has significant economic implications for both healthcare systems and patients. Some studies suggest that annual diabetes management costs can be 5.4 times higher in patients with DFUs and may increase to 8 times higher in severe cases [16].

Despite warnings about the need for early detection and treatment, prevention efforts remain inadequate, with inconsistent follow-up and patient compliance [17,18]. Consequently, individuals with DFUs often have a reduced quality of life, higher rates of depression, and a 5-year mortality rate of up to 74% [19,20].

Our study examined various laboratory data, starting with routine blood tests, to identify risk factors associated with the need for surgical amputation in diabetic foot cases. We aim to identify risk factors that can be modified from reversible conditions to enhance clinical decision-making, thereby offering patients potential alternatives to lower limb amputation.

## 2. Materials and Methods

A retrospective case–control study according to the STROBE guidelines [21] was conducted.

This study included patients older than age 18 who had required lower extremity amputation from January 2017 to April 2021. Patients with lower extremity amputation due to causes different from DF complications were excluded. Cases were defined as the patients who were evaluated in the 5 days before surgery. Controls were the patients who were evaluated 30 days before surgery. Case–control matching was carried out according to age, sex, and number of amputation surgeries. The expected follow-up period for the cases was 3 years.

We identified 414 patients taken consecutively in chronological order from the earliest first surgery, of which 200 fulfilled the inclusion and exclusion criteria and were divided into 2 groups: 100 patients in the group of cases (evaluated in the 5 days before surgery) and 100 ones in the group of controls (evaluated 30 days before surgery).

The assessed variables included age, sex, reamputation, hemoglobin (Hb), white blood count (WBC), neutrophil-to-lymphocyte ratio (NLR), platelet-to-lymphocyte ratio (PLR), C-reactive protein (CRP), and procalcitonin.

All the patients analyzed in this study were hospitalized and surgically treated in the Orthopedics and Traumatology Department of the Fondazione Policlinico Universitario A. Gemelli IRCCS in Rome. 

This study was conducted in accordance with the ethical standards outlined in the 1964 Declaration of Helsinki, and informed consent to the processing of data was obtained from all patients.

### Statistical Methods

Descriptive statistics were used. For continuous variables, central tendency and dispersion measures were used. Statistical significance was calculated using Student’s *t*-test.

Our analysis identifies associations between the assessed variables and the time before the first surgical amputation of lower limb rather than predictive variables.

A multiple linear regression analysis was performed to assess whether age and sex acted as confounders in the relationship with blood test variables and 3-year mortality.

All analyses were carried out using the SPSS statistical package, version 29.0 for Windows (SPSS, Chicago, IL, USA). All tests used a 0.05 statistical significance level with the statistical software.

## 3. Results

### 3.1. Patient Characteristics

Sociodemographic characteristics are shown in Table 1.

### 3.2. Hb, WBC, NLR, PLR, CRP, and Procalcitonin

The average hemoglobin values were lower in the group of patients evaluated within 5 days before surgery (10.12 mg/dL in cases and 11.62 mg/dL in controls).

The average WBC values were higher in the group of patients evaluated within 5 days before surgery (11.16 × 10^9^ /L in cases and 10.96 × 10^9^ /L in controls), as NLR (5.07 in cases and 4.14 in controls), PLR (193.28 in cases and 165.42 in controls), and CRP (100.18 mg/dL in cases and 93.68 mg/dL in controls).

The average procalcitonin values were higher in the group of patients evaluated within 30 days before surgery (0.12 ng/mL in cases and 0.16 ng/mL in controls).

The reduction of Hb, as the increases of NLR, PLR, and CRP in the cases were statistically significant (*p* = 0.001) (Table 2). The increases of WBC, as the reduction of procalcitonin in the cases were not statistically significant (*p* = 0.414, and *p* = 0.450 respectively).

### 3.3. Follow Up

Among the 100 patients of the group of the cases, we investigated the survival at 3 years from the first amputation surgery. A total of 44 patients survived after 3 years from the first amputation surgery, 33 died, and we had no data available about 23 patients.

The patients who survived after 3 years from the first amputation surgery had a lower average age than the patients who passed within 3 years (63.84 years old versus 74.18 years old), and the difference was statistically significant (*p* = 0.001) (Table 3).

There was a prevalence of male patients in the followed-up group, with no statistically difference among the 3-year survived and dead ones.

Most followed-up patients underwent a single amputation surgery: 30 amputated and 14 reamputated surviving patients; 27 amputated and 6 reamputated deceased patients. The difference in the number of surgeries was not statistically significant in the two groups.

The average Hb values were lower and not statistically significant in the group of followed-up patients who died within 3 years from the first surgery.

The average WBC values were higher in the group of followed-up patients who died within 3 years from the first surgery (9.99 × 10^9^ /L in 3-year follow-up survivors and 12.40 × 10^9^ /L in 3-year follow-up deaths), as NLR (4.89 in 3-year follow-up survivors and 6.84 in 3-year follow-up deaths), PLR (204.34 in 3-year follow-up survivors and 209.81 in 3-year follow-up deaths), and CRP (95.33 mg/dL in 3-year follow-up survivors and 146.35 mg/dL in 3-year follow-up deaths). Each value was statistically significant in the two groups (Table 4).

The average procalcitonin values were higher and not statistically significant in the group of followed-up patients who survived the 3 years following the first surgery.

### 3.4. Confounding Factors

The multiple linear regression analysis aimed to determine whether age and sex independently contributed to blood test levels and 3-year mortality, as well as to assess their potential confounding effects. Regression coefficients and *p*-values were reported (Table 5). The analysis was not significant, with *p*-values greater than 0.05. Furthermore, when comparing the coefficient of the independent variable with and without the inclusion of sex and age in the model, no change greater than 10% was observed. This suggests that sex and age may not be confounders.

## 4. Discussion

This study demonstrated a statistically significant correlation between some laboratory test values and the need to perform a surgical amputation of part of the lower limb within the time span of 30 days to treat the patients affected by diabetic foot disease.

In particular, we demonstrated a statistically significant correlation between the reduction in Hb values, as well as increases in NLR, PLR, and CRP values and the need to treat patients affected by diabetic foot disease with amputation surgery.

Anemia is frequently reported as a complication of DM [22]. It has been linked to delayed wound healing, an increased risk of amputation, and heightened mortality rates [23]. Recent research indicates that anemia is particularly common in DM patients with diabetic foot ulcers (DFUs) [24,25]. A meta-analysis demonstrated a positive correlation between the severity of anemia and DFU severity, suggesting anemia’s potential as a predictor for amputation and mortality [26]. Retrospective studies support that anemia is associated with more extensive ulcers, severe infections, and higher risks of both amputation and mortality [27,28]. Additionally, observational studies in Nigeria have associated anemia with delayed wound healing, amputation, and higher mortality rates [23,29]. Recent research from Korea found that Hb levels were notably lower in diabetic foot patients than in healthy individuals, especially in those over 60 or hospitalized for over two weeks [30]. Addressing anemia is, therefore, essential for enhancing wound healing in diabetic foot patients.

The NLR is an economical and accessible biomarker, valuable for evaluating prognosis in various inflammatory, viral, cardiovascular, and cancerous conditions [31,32,33,34]. Unlike specific wound markers like matrix metalloproteinases or growth factors, NLR offers a practical approach to assessing immune activity at a lower cost. It is also stable and resilient to changes influenced by dehydration, exercise, or blood processing, factors that can affect other biomarkers [35]. Numerous studies underscored its relevance in systemic inflammation within diabetes [36,37].

The PLR, like NLR, can be readily assessed and has shown predictive value for DM and its complications [38,39,40]. Zhang et al. found significantly elevated PLR levels in patients with DFUs, identifying it as an independent risk factor [41]. According to Demirdal et al., higher PLR levels were linked to osteomyelitis and served as a predictive marker for amputation in diabetic foot infections [42]. Elevated PLR in this study may result from increased platelet (PLT) counts, signifying a heightened inflammatory and thrombotic state, along with a decline in lymphocytes (LYM) due to inflammation’s effects. PLR, which combines these two indicators, thus provides a more comprehensive correlation with DFU. However, its precise role requires further investigation.

CRP rises in response to inflammation, infection, tissue trauma, and various diseases. Although non-specific, CRP serves as a useful indicator for managing diabetic foot disease, as a rapid drop in CRP suggests effective treatment response [43]. Baseline acute-phase reactants correlate with increased amputation risk, with baseline CRP levels showing predictive value for both overall and major amputations. Conversely, the baseline erythrocyte sedimentation rate (ESR) did not show similar predictive capacity, as CRP levels tend to decrease more rapidly after treatment, indicating effectiveness more sensitively than ESR [44]. Volaco et al. similarly reported that elevated CRP levels are strong predictors of major amputation in long-term diabetic patients with ischemic foot conditions [45]. Lipsky et al. found that elevated baseline levels of WBC, CRP, and ESR were linked to treatment failure in diabetic foot infections managed with broad-spectrum antibiotics [43]. In alignment with current literature, our findings confirmed the correlation between CRP levels and surgical amputation in diabetic foot cases.

Our study also revealed a significant negative correlation between older age at the time of initial surgical amputation and three-year survival post-amputation in diabetic foot cases. Aging is often accompanied by cumulative pathologies, and as noted by Crispin et al., several joint and bone conditions are associated with diabetes mellitus [46]. Specifically, neuropathic arthropathy affects around 0.1% to 0.4% of diabetic patients, particularly between ages 50 and 69, with no observed gender differences; in 20% of cases, both sides are affected. The most impacted joints include metatarsophalangeal, tarsometatarsal, tarsus, ankle, and interphalangeal, whereas knee, wrist, elbow, shoulder, and intervertebral joints are less frequently involved [47].

Our data also indicated a significant correlation between lower WBC, NLR, PLR, and CRP values at the initial amputation date and three-year survival rates. Consistent with numerous studies, NLR, PLR, and CRP serve as negative prognostic markers in DFU patients [48,49,50,51]. No studies are currently present in the literature explaining a relation between WBC and its prognostic role in diabetic foot disease. No study demonstrated which of these indices may be more useful in clinical use.

A limitation of this study is its retrospective case–control design, which does not allow for the determination of causality between laboratory parameters and amputation risk in patients with diabetic foot disease. This design is also subject to potential biases in the selection of cases and controls, as well as in the collection of retrospective data. A prospective multicenter cohort study, in contrast, would have enabled us to directly follow patients over time to assess the relationship between laboratory variations and outcomes and to evaluate how these variations might affect the need for amputation surgery compared to other treatments. Such a design would also allow for a comparison of the 3-year survival rates between patients treated with amputation surgery and those treated with alternative methods. Although longitudinal data over the 3-year follow-up period were not included in this retrospective study, tracking these parameters over time could yield valuable insights into their evolution and their ability to predict worsening outcomes or increased amputation risk. We highlight the potential value of such longitudinal data, and we recommend that future studies explore biomarker fluctuations in relation to clinical outcomes.

Moreover, hematological parameters can be influenced by underlying conditions, such as anemia and its differential diagnoses [52]. A limitation of this study was interpreting these parameters independently, not considering other comorbid conditions (e.g., cardiovascular disease, renal impairment), and we suggest that future studies account for these potential confounding factors.

Furthermore, while NLR, PLR, and CRP levels were significantly associated with amputation risk, a regression model should be needed to establish a predictive role. We recommend further studies that incorporate regression analysis to evaluate NLR, PLR, and CRP predictive potentials.

This study did not directly assess diabetes-specific markers, such as HbA1c levels or insulin resistance indicators. We highlight the importance of including such markers in future studies to capture a more comprehensive view of factors associated with diabetic foot outcomes.

The presence of myeloproliferative disorders in the study sample was not considered. Since they are highly regarded as thrombogenic pathologies [53], we acknowledge the role of these disorders as potential confounding factors in the association between hematological parameters and amputation risk

Despite the low specificity of inflammatory markers assessed, they are widely used in clinical practice to indicate general inflammatory status, especially in cases where specific markers are not available [54].

In this study, we focused on exploring associations rather than identifying specific predictive thresholds with calculated sensitivity and specificity values. These findings should be interpreted with caution due to limitations inherent in the case–control design. We suggest the establishment of parameter-specific cut-offs in future studies for enhanced predictive accuracy. Furthermore, we believe that the use of scoring systems based on these parameters could represent an interesting development for future studies aimed at predicting the risk of amputation.

## 5. Conclusions

Our findings suggested that lower Hb levels and elevated CRP, NLR, and PLR levels in patients with diabetic foot disease may be associated with a higher risk of lower-limb amputation. Unexpectedly, this study also demonstrated that older age, WBC, CRP, NLR, and PLR levels may be associated with a negative prognosis for these patients. These findings highlighted the importance of monitoring these laboratory values in diabetic patients in identifying individuals at higher risk of leg amputations and implemented appropriate interventions to prevent or minimize the occurrence of these severe complications.

## Figures and Tables

**Table 1:** Patient characteristics.

	Evaluated at 30 Days Before Surgery (n = 100)	Evaluated Within 5 Days Before Surgery (n = 100)	Total (n = 200)
Age (years)			
Average	67.6	67.69	67.64
(SD)	10.71	11.78	11.23
Median	68	67	67.5
(Q1–Q3)	(59–76)	(61–75)	(60–76)
(min–max)	(44–91)	(34–95)	(34–95)
Sex			
Male	75 (75%)	74 (74%)	149 (74.5%)
Female	25 (25%)	26 (26%)	51 (25.5%)
Number of surgeries			
Amputation	83 (83%)	78 (77%)	161 (80.5%)
Reamputation	17 (17%)	22 (23%)	39 (19.5%)

SD (standard deviation).

**Table 2:** Hematological parameters according to time span before the first amputation surgery.

	Evaluated at 30 Days Before Surgery (n = 100)	Evaluated Within 5 Days Before Surgery (n = 100)	Total (n = 200)	*p*-Value
Hb (mg/dL)				0.001 *
Average	11.62	10.12	10.87	
(SD)	1.59	1.54	1.56	
Median	11.7	9.9	10.8	
(Q1–Q3)	(10.8–12.7)	(9–10.95)	(9.9–11.82)	
(min–max)	(7.9–15.6)	(7.3–13.9)	(7.3–15.6)	
WBC (×10^9^/L)				0.414
Average	10.96	11.16	11.06	
(SD)	5.29	5.95	5.62	
Median	9.42	9.54	9.48	
(Q1–Q3)	(7.48–13.36)	(7.7–13.09)	(7.59–13.22)	
(min–max)	(3.11–36.83)	(3–38.79)	(3.11–38.79)	
NLR				0.001 *
Average	4.14	5.07	4.6	
(SD)	2.45	3.25	2.85	
Median	3.68	3.94	3.81	
(Q1–Q3)	(2.22–5.46)	(2.56–7.06)	(2.39–6.26)	
(min–max)	(0.14–10.33)	(0.69–13.94)	(0.14–13.94)	
PLR				0.001 *
Average	165.42	193.28	179.35	
(SD)	86.35	94.65	90.5	
Median	148.64	179.91	164.27	
(Q1–Q3)	(105.97–220.37)	(125.15–251.35)	(115.56–235.86)	
(min–max)	(2.01–394)	(2.67–437.28)	(2.01–437.28)	
CRP (mg/dL)				0.001 *
Average	93.68	100.18	96.93	
(SD)	82.34	81.9	82.12	
Median	72.7	73.3	73	
(Q1–Q3)	(22.27–142.58)	(32.2–151.1)	(27.23–146.84)	
(min–max)	(0.7–326.9)	(0.5–320.8)	(0.5–326.9)	
Procalcitonin (ng/mL)				0.450
Average	0.16	0.12	0.14	
(SD)	0.12	0.08	0.1	
Median	0.12	0.09	0.1	
(Q1–Q3)	(0.05–0.23)	(0.05–0.18)	(0.05–0.2)	
(min–max)	(0.05–0.49)	(0.31–0.49)	(0.05–0.49)	

* Statistically significant; Hb (hemoglobin); WBC (white blood count); NLR (neutrophil-to-lymphocyte ratio); PLR (platelet-to-lymphocyte ratio); CRP (C-reactive protein); SD (standard deviation).

**Table 3:** Patient characteristics according to survival rate at 3 years from the first amputation surgery.

	3-Year Survivors (n = 44)	3-Year Deaths (n = 33)	Total (n = 77)	*p*-Value
Age (years)				0.001 *
Average	63.84	74.18	68.14	
(SD)	11.28	8.6	11.41	
Median	63.5	74	68	
(Q1–Q3)	(57.25–71)	(66–81.25)	(60.75–76)	
(min–max)	(34–93)	(56–91)	(34–93)	
Sex				0.507
Male	36 (81.82%)	24 (72.73%)	60 (77.92%)	
Female	8 (18.18%)	9 (27.27%)	17 (22.08%)	
Number of surgeries				0.402
Amputation	30 (68.18%)	27 (81.82%)	57 (74.03%)	
Reamputation	14 (31.82%)	6 (18.18%)	20 (25.97%)	

* Statistically significant; SD (standard deviation).

**Table 4:** Hematological parameters according to survival rate at 3 years from the first amputation surgery.

	3-Year Survivors (n = 44)	3-Year Deaths (n = 33)	Total (n = 77)	*p*-Value
Hb (mg/dL)				0.368
Average	10.58	10.3	10.46	
(SD)	1.87	1.57	1.74	
Median	10.05	9.9	10	
(Q1–Q3)	(9.17–11.3)	(9.5–10.65)	(9.4–11.3)	
(min–max)	(7.9–15.7)	(7.9–13.3)	(7.9–15.7)	
WBC (×10^9^/L)				0.001 *
Average	9.99	12.40	11.02	
(SD)	4.84	6.14	5.52	
Median	9.08	11.5	10.03	
(Q1–Q3)	(7.62–11.38)	(8.59–15.5)	(7.98–12.82)	
(min–max)	(0.33–24.13)	(0.62–26.14)	(0.33–26.14)	
NLR				0.001 *
Average	4.89	6.48	5.53	
(SD)	3.18	3.63	3.43	
Median	3.94	6.06	4.54	
(Q1–Q3)	(2.57–6.21)	(3.33–8.95)	(2.88–7.72)	
(min–max)	(1.5–12.72)	(1.29–13.27)	(1.29–13.27)	
PLR				0.034 *
Average	204.34	209.81	206.58	
(SD)	95.76	91	93.11	
Median	180.68	187.25	185.24	
(Q1–Q3)	(146.15–271.46)	(140.15–264.32)	(142–267.94)	
(min–max)	(4.21–437.28)	(76.92–406.48)	(4.21–437.28)	
CRP (mg/dL)				0.001 *
Average	95.33	146.35	114.46	
(SD)	98.96	96.67	100.36	
Median	67.9	123.5	100.35	
(Q1–Q3)	(20.65–129.3)	(75.5–215)	(26.12–149.82)	
(min–max)	(1.2–407.2)	(16–317.4)	(1.2–407.2)	
Procalcitonin (ng/mL)				0.940
Average	0.15	0.13	0.14	
(SD)	0.12	0.11	0.11	
Median	0.08	0.11	0.09	
(Q1–Q3)	(0.05–0.23)	(0.06–0.14)	(0.05–0.21)	
(min–max)	(0.05–0.42)	(0.05–0.4)	(0.05–0.42)	

* Statistically significant; Hb (hemoglobin); WBC (white blood count); NLR (neutrophil-to-lymphocyte ratio); PLR (platelet-to-lymphocyte ratio); CRP (C-reactive protein); SD (standard deviation).

**Table 5:** Multiple linear regression analysis for sex and age as confounding factors.

	Coefficient for Sex (*p*-Value)	Coefficient for Age (*p*-Value)
Hb 30 days before surgery	0.64 (0.08)	−0.01 (0.72)
Hb within 5 days of surgery	0.31 (0.21)	−0.01 (0.23)
WBC 30 days before surgery	−1.13 (0.17)	−0.05 (0.14)
WBC within 5 days of surgery	−1.49 (0.12)	0.05 (0.18)
NLR 30 days before surgery	0.15 (0.67)	0.01 (0.65)
NLR within 5 days of surgery	−0.27 (0.59)	0.03 (0.11)
PLR 30 days before surgery	7.26 (0.57)	−0.18 (0.72)
PLR within 5 days of surgery	0.38 (0.98)	0.51 (0.37)
CRP 30 days before surgery	−18.82 (0.11)	−0.29 (0.53)
CRP within 5 days of surgery	−12.1 (0.32)	0.56 (0.24)
Procalcitonin 30 days before surgery	0.01 (0.67)	0.01 (0.25)
Procalcitonin within 5 days of surgery	−0.01 (0.25)	−0.01 (0.77)
3-year mortality	0.21 (0.08)	−0.01 (0.06)

Hb (hemoglobin); WBC (white blood count); NLR (neutrophil-to-lymphocyte ratio); PLR (platelet-to-lymphocyte ratio); CRP (C-reactive protein).

## Data Availability

The data presented in this study are available upon request from the corresponding author due to privacy.
